# Consumer Perception of Irradiated Food Products in the Abai Region of Kazakhstan

**DOI:** 10.3390/foods14091625

**Published:** 2025-05-04

**Authors:** Duman Orynbekov, Zhanar Kalibekkyzy, Almagul Nurgazezova, Gulnur Nurymkhan, Amirzhan Kassenov, Yernaz Yermekov

**Affiliations:** 1 Department of Food Production Technology and Biotechnology, The Engineering-Technological Faculty, Shakarim University, Glinka 20A, Semey 071412, Kazakhstan; zhanar_moldabaeva@mail.ru (Z.K.); almanya1975@mail.ru (A.N.); gulnu-n@mail.ru (G.N.); 2 Department of Food Technology and Processing Products, Technical Faculty, Saken Seifullin Kazakh Agrotechnical Research University, Zhenis Avenue, 62, Astana 010011, Kazakhstan; amirzhan-1@mail.ru

**Keywords:** food irradiation, consumer attitudes, food safety, Kazakhstan, knowledge gap, technology acceptance

## Abstract

Food irradiation is a scientifically validated method for improving food safety and shelf life, yet public acceptance remains limited due to persistent misconceptions. This study investigates consumer perceptions of irradiated food in the Abai region of Kazakhstan, an area historically affected by radiation exposure, though this context is not explicitly addressed. A structured questionnaire was administered to 420 adult residents across urban and rural districts, collecting data on familiarity, knowledge, perceived risks and benefits, willingness to consume, and influencing factors such as trust in authorities and preference for natural foods. Descriptive and inferential statistical analyses, including Pearson correlation and Duncan’s test, were employed. Results reveal widespread knowledge gaps—only 20% correctly rejected the myth that irradiated food is radioactive—yet nearly half of respondents expressed willingness to purchase such products. Perceived health risk was high across all food categories, especially infant food, but this did not consistently predict avoidance. Demographic variables such as education and age influenced perceptions of necessity, while gender and trust in authorities had minimal impact. The study concludes that although consumer knowledge is low, moderate openness exists toward irradiated food, suggesting that public education and clear communication could play a pivotal role in building acceptance for this food safety intervention.

## 1. Introduction

Food irradiation is a food safety intervention that involves exposing food to controlled levels of ionizing radiation (e.g., gamma rays, X-rays, or electron beams) to destroy harmful bacteria, parasites, and insects, thereby extending shelf life and reducing the risk of foodborne illness [1,2,3,4,5,6,7]. The process has been endorsed by the World Health Organization since 1980 as a safe and effective technology for improving food hygiene [8]. Importantly, irradiating food does not make it radioactive; the energy levels used are sufficient to inactivate microorganisms but do not induce residual radioactivity in the food. Many countries have approved food irradiation for products such as spices, dried herbs, fresh produce, and meats as a means to ensure quarantine security and reduce pathogens. Despite its scientific validation and practical benefits, consumer acceptance of irradiated foods has historically been a challenge. Misconceptions—such as the belief that irradiated food becomes radioactive or less nutritious—have been common and can evoke public concern [9,10,11,12,13,14].

Global consumer attitudes toward irradiated foods are gradually evolving. A recent systematic review found that worldwide acceptance of irradiated food has increased significantly over the past few decades, from about 33% of consumers willing to purchase in 1992 to roughly 67% in 2024 [15]. During the same period, familiarity with the concept of food irradiation remained around 50% globally, indicating that while more people are willing to consider irradiated foods, overall public awareness has not grown commensurately. Even in countries where food irradiation has been discussed for years, many consumers still lack a clear understanding of what the process entails. For example, a 1995 U.S. survey reported that 72% of respondents had heard of food irradiation, but among those, 87% admitted they “do not really know much about it” [9]. In that study, over 30% of consumers believed irradiated food is radioactive, underscoring how persistent this key misconception can be. On a positive note, consumers were found to be less worried about irradiation than about several other food safety issues (such as pesticide residues, additives, or bacterial contamination), ranking irradiation as a comparatively lower concern. Around 45% of those U.S. consumers said they would buy irradiated food if available (19% said they would not), suggesting nearly half the public could accept the technology given proper assurances [15].

Public acceptance often hinges on trust and understanding. If consumers are well-informed about the nature and benefits of food irradiation, their attitudes tend to become more favorable [2,13,16]. Effective communication—highlighting that irradiation can prevent illness (by killing *E. coli*, *Salmonella*, etc.), reduce spoilage, and is endorsed by health authorities—has been shown to significantly improve willingness to purchase irradiated foods [11,17,18,19]. Conversely, lack of information or negative associations can dampen acceptance. The term “irradiation” itself may evoke fear, especially in populations with historical experiences related to radiation exposure. Kazakhstan provides a unique context in this regard. The Abai region (formerly part of Semipalatinsk area in East Kazakhstan) was the primary site of Soviet nuclear weapons testing for decades. As a result, radiation and its health effects are an emotive subject in Kazakhstan [20],

The Semipalatinsk Nuclear Test Site (SNTS), located in eastern Kazakhstan, was the principal Soviet testing ground for nuclear weapons between 1949 and 1989 [21]. During this period, 459 nuclear detonations were conducted [22]—atmospheric, underground, and surface—resulting in widespread radiological contamination [23]. With a cumulative explosive yield approximately 2500 times that of the Hiroshima bomb, the health and environmental consequences for surrounding populations were profound [24,25,26]. Epidemiological studies have documented elevated rates of cancer [27,28,29,30], congenital disorders [31,32], and transgenerational health effects among residents in the vicinity [32], with over 1.5 million people affected to varying degrees [32].

In 1991, Kazakhstan became the first nation to voluntarily renounce nuclear weapons infrastructure, formally closing the SNTS by presidential decree [33]. Despite the cessation of tests, the legacy of nuclear exposure remains a salient issue in Kazakh society—particularly in the Abai region [20], where the population continues to live with the psychological and biological aftermath of prolonged radiation exposure.

Although food irradiation is fundamentally distinct from nuclear testing—employing controlled, non-residual ionizing radiation to improve microbial safety without leaving radioactivity—the association with the term “radiation” can elicit deep-seated public apprehension. The psychological imprint left by decades of nuclear testing may influence consumer attitudes, leading to confusion, mistrust, or rejection of otherwise safe technologies. This historical legacy might influence how consumers perceive anything labeled “irradiated”. On the one hand, people in affected communities might be more apprehensive about technologies involving radiation; on the other hand, they may also be more aware of radiation issues and therefore potentially open to learning the distinctions between nuclear fallout and controlled food irradiation. To date, however, there has been little to no published research on Kazakhstani consumers’ perceptions of irradiated food, and no irradiated products labeled as such are present on the market.

Understanding local consumer perception is crucial as Kazakhstan considers modern food processing technologies. The country is a major producer and exporter of agricultural products in Central Asia [34], and implementing irradiation for example, to meet phytosanitary standards [35] or reduce post-harvest losses [36], could bring economic and public health benefits [37]. Indeed, Kazakhstan has established regulations permitting food irradiation for certain purposes, e.g., a CIS guideline GOST 33302–2015 for phytosanitary treatment of produce [38]. Irradiation is widely used worldwide for export commodities: for instance, the United States irradiates roughly 120,000 tons of food annually, most of them being spices, pet food and ground beef. Asia treats 285,223 tons of food products annually, of which China treats around 200,000 tons with 100 functioning irradiators [2]. Spices and dried herbs are a common category for irradiation, accounting for a large share of the global irradiated food volume [39]. For Kazakhstan, which exports and produces various grains [40], meats [41], and produce [42], adopting irradiation for spices or quarantine treatment of export goods could help meet international safety requirements. Any move to introduce irradiated foods into the consumer market must address public opinion and concerns. Lessons from other countries indicate that consumer education, even minimal [14], and transparent communication [43] are vital. When consumers are given clear, accessible explanations and can weigh the *immediate benefits* (such as reduced food poisoning risk or longer-lasting fresh foods), acceptance tends to increase [2,44,45,46]. In contrast, if misconceptions go uncorrected [9] or if trust in authorities is low [47], consumers may reject irradiated foods even if they are safer.

Given these sensitivities and perspectives of the irradiation technology, the present study investigates consumer perception of irradiated food products in the Abai region, where the influence of the SNTS legacy is most pronounced. The study explores familiarity with food irradiation, knowledge accuracy, risk perception, and willingness to consume such products. By assessing these factors in a population uniquely shaped by historical exposure to radiation, this research contributes valuable insight into context-dependent acceptance of food technologies and informs strategies for risk communication and public engagement in post-nuclear regions.

## 2. Materials and Methods

### 2.1. Study Design and Sample

**Figure 1:**
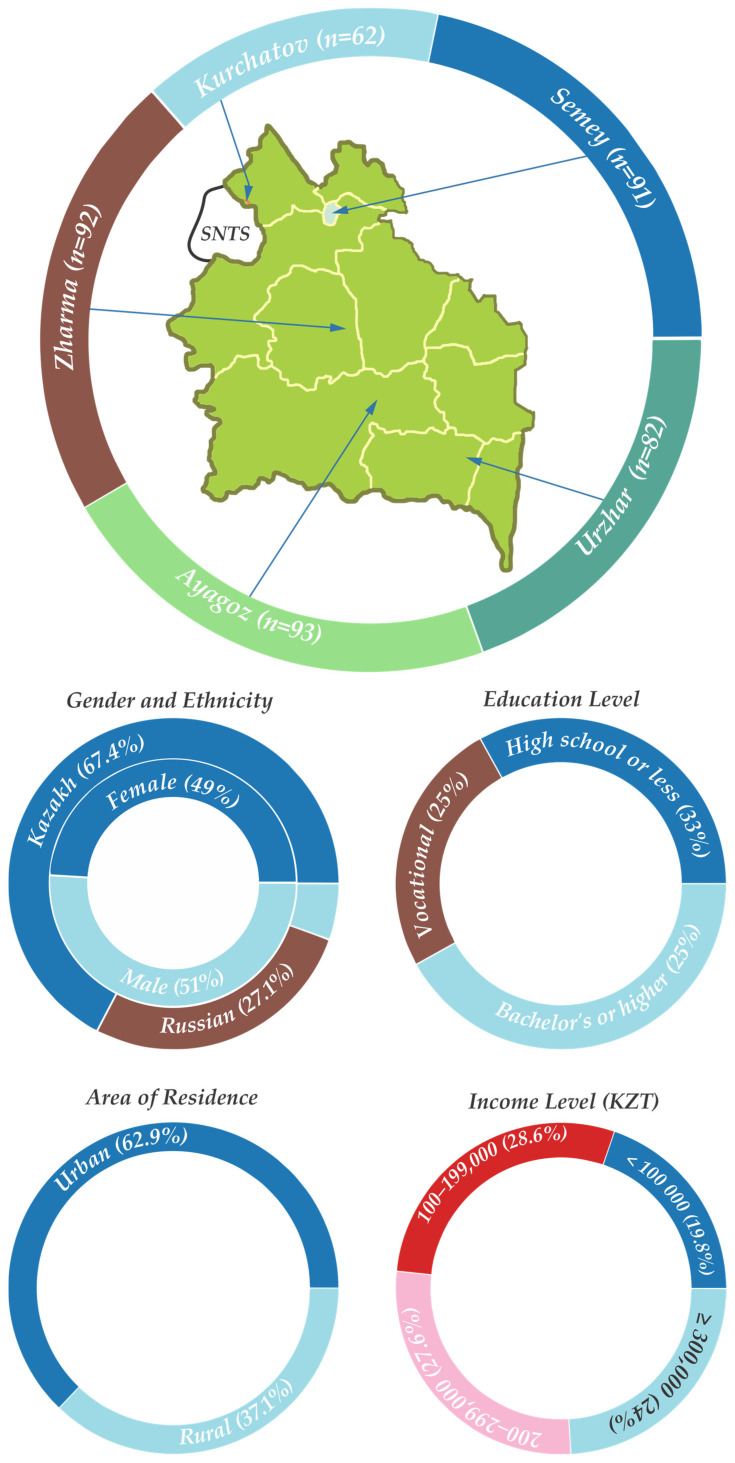
Sample characteristics.

A cross-sectional survey was conducted in the Abai region of eastern Kazakhstan to collect data on consumer perceptions of food irradiation. The target population was adult consumers, aged 18 and above, residing in various districts of the region. A total of 420 respondents were recruited from five key localities: the city of Semey (the regional center), and the towns of Ayagoz, Zharma, Urzhar, and Kurchatov (Figure 1). Figure 1 shows the relative locations of urban and rural population centers to SNTS and the demographic data of the sample.

These locations were chosen to provide a mix of urban and rural perspectives, as well as to capture any differences that might arise from proximity to the former Semipalatinsk nuclear test site (e.g., Kurchatov is located near the test site, Urzhar being the furthest away). The sample included 264 urban residents (62.9%) and 156 rural residents (37.1%), roughly reflecting the region’s urban–rural split. Within each location, participants were selected through convenience sampling at central marketplaces, shopping areas, and community gathering points. The survey was administered in person by trained interviewers in the Kazakh or Russian languages (according to the participant’s preference). Participation was voluntary and anonymous; respondents gave oral and written signed informed consent after being briefed on the purpose of the study. The study protocol was reviewed for ethical compliance, and sensitive questions (such as income) were optional to answer.

#### Questionnaire

The questionnaire was developed specifically for this study, drawing on items used in previous consumer research on irradiated foods [1,9], and adapted to the Kazakhstani context. It consisted of multiple sections covering demographic information, awareness and knowledge, risk perceptions, acceptance, and related attitudes. Overview of the questionnaire is given in Table 1.

The questionnaire was developed in Kazakh and Russian. It was reviewed by bilingual experts to ensure conceptual equivalence. A pilot test with 10 individuals from the target region was conducted to check clarity and timing, leading to minor wording adjustments. Each survey interview took approximately 15–20 min to complete.

### 2.2. Statistical Analysis of Data

Survey responses were coded and entered into a spreadsheet for analysis. Descriptive statistics were computed for all variables. Categorical variables (e.g., familiarity levels, yes/no sources, responses to knowledge and attitude questions) are summarized as frequencies and percentages. Continuous or ordinal variables like age (treated as continuous) and Likert-scale responses (treated as ordinal scales) are summarized with means and standard deviations or medians as appropriate. Key descriptive findings are presented in tabular form.

Survey responses were coded and entered into a spreadsheet for analysis. Descriptive statistics were computed for all variables. Categorical variables (e.g., familiarity levels, yes/no information sources, responses to knowledge and attitude questions) are summarized as frequencies and percentages. Continuous or ordinal variables like age (treated as continuous) and Likert-scale responses (treated as ordinal scales) are summarized with means and standard deviations or medians as appropriate. Key descriptive findings are presented in tabular form (see Section 3).

For bivariate analyses, we employed both association tests and comparative tests. Pearson’s chi-square tests were used to test for associations between categorical variables. For example, we used chi-square to assess if willingness to buy irradiated food (categorized as likely/neutral/unlikely) differed by gender, age group, education level, etc. Chi-square was also used to see if there were significant differences in response distributions for items like perceived necessity across different demographic subgroups.

Pearson correlation analysis was performed among several scale variables (after assigning numeric codes) to evaluate linear relationships. We computed a correlation matrix including variables such as: familiarity level (on a 1–5 scale), total knowledge score (count of correct answers out of 5), risk perception index (averaged across items, with Likely = 3, Neutral = 2, Unlikely = 1 for each category), willingness/acceptance index (averaged across the four food categories, coded 3 for likely, 2 for neutral, 1 for unlikely), and the three attitudinal measures (each coded 1–5). This allowed us to check, for instance, whether greater knowledge correlates with lower perceived risk or higher willingness, and whether general trust or technophilia correlates with acceptance.

For group comparisons, if variables were measured at an ordinal or approximately interval level, we conducted one-way analysis of variance (ANOVA) or a nonparametric equivalent (Kruskal–Wallis) when appropriate. For example, we tested whether the mean knowledge score differed across education levels, or whether mean perceived necessity ratings differed by demographic categories. In cases where a significant overall difference was found (*p* < 0.05), we applied Duncan’s multiple range test as a post hoc analysis to identify which specific groups differed. Duncan’s test is a multiple comparison procedure similar to Tukey’s HSD but slightly more liberal in detecting differences; it was chosen here to explore patterns in the data (such as between different education groups) where an ordered trend might exist.

In analyzing differences between food categories within the same respondents (e.g., is perceived risk higher for one type of food than another?), we treated the responses as related observations. We used the Friedman test (a nonparametric test for related samples) to check if there were overall differences in distributions across food categories, since the perception data were ordinal. If notable differences were observed, they are reported; however, most such within-respondent comparisons did not reach statistical significance.

All statistical tests were two-tailed with a significance threshold of α = 0.05. Data analysis was performed using IBM SPSS Statistics 26 Because of the largely descriptive and exploratory nature of the study, we focus on observed patterns and effect sizes; exact *p*-values are reported for key findings. The results below are organized following the logical sequence of the survey: first demographics and awareness, then knowledge, then risk and acceptance attitudes, followed by factors influencing acceptance.

## 3. Results

### 3.1. Sample Characteristics

A total of 420 consumers participated in the survey. Table 2 summarizes their demographic profile and provides the data from the national survey conducted by the Bureau of National Statistics of Kazakhstan in 2021 as well. The sample was almost evenly split by gender (51.0% female, 49.0% male). Ages ranged from 18 to 79 years, with a mean age of approximately 49.6 (±18.6) years. Just over one-third of respondents (33.3%) were aged 60 or older, about 19% were younger adults under 30, and the remainder were middle-aged. The two predominant ethnic groups were Kazakh (67.4% of respondents) and Russian (27.1%), reflecting the region’s population; a small fraction (5.5%) identified as other ethnicities (such as Tatar, Ukrainian, etc.). In terms of education, 41.9% had a bachelor’s degree or higher, 25.0% had some college or vocational training, and 33.1% had a high school education or less. Monthly income levels varied: roughly one-fifth reported household income below 100,000 KZT per month, while about one-quarter earned 300,000 KZT or more per month, with the rest in intermediate ranges. Just under two-thirds of the sample (62.9%) lived in urban areas (the cities of Semey or Ayagoz), and 37.1% lived in rural districts or villages. All five targeted localities contributed substantially to the sample (each around 15–22% of respondents), ensuring geographic diversity within the Abai region.

Overall, the sample represents a broad cross-section of the Abai region’s consumer population in terms of age, gender, and socio-economic background. Any deviations from the region’s exact population proportions (e.g., perhaps a slight overrepresentation of more educated individuals, given 42% had a bachelor’s degree) are acknowledged as a limitation of convenience sampling like accessibility and willingness to participate. While efforts were made to include diverse demographic groups, the sampling approach may introduce bias and limit the generalizability of the findings. A comparative overview with national demographic statistics is presented in Table 1 to support the contextual understanding of sample representativeness, as the national survey conducted by the National Bureau of Statistics includes all age groups in the income bracket data, the lower income bracket is disproportionately overrepresented in the Country level data. The diversity captured allows for a meaningful analysis of how perceptions might differ across these demographic segments.

### 3.2. Awareness and Familiarity with Food Irradiation

**Figure 2:**
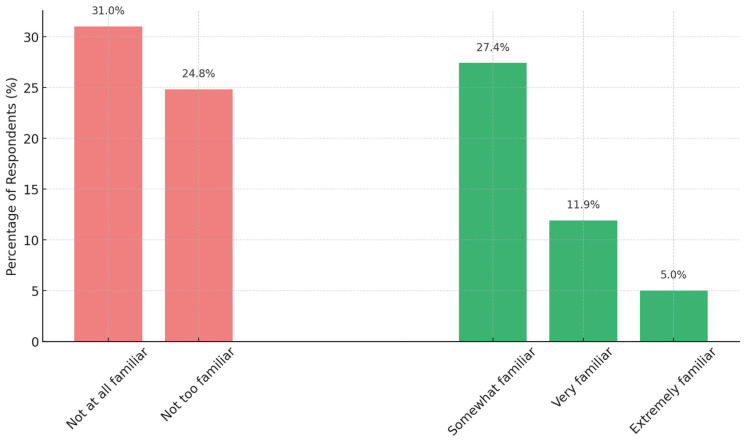
Respondents’ familiarity with food irradiation by category (N = 420).

One of the first aims was to determine how familiar consumers are with the concept of food irradiation. When asked to rate their familiarity, only a small minority claimed to be “Very familiar” or “Extremely familiar”. Specifically, as shown in Figure 2, just 16.9% of respondents described themselves as either “Very familiar” (11.9%) or “Extremely familiar” (5.0%) with food irradiation. On the other end of the spectrum, about 31.0% admitted they were “Not at all familiar” with it, meaning they had never heard of the concept. The remaining respondents were in between: 24.8% said “Not too familiar” and 27.4% said “Somewhat familiar”. If we consider those answering “Somewhat” or above as having at least some awareness, roughly 50–55% of the sample had heard of food irradiation to some extent. In other words, about half of consumers in Abai region have at least heard of food irradiation, while the other half are essentially unaware of it. This awareness level is comparable to global estimates (around 50% familiarity) noted in other studies, but there is clearly a wide range in depth of understanding.

For those who had at least heard of food irradiation, the survey inquired about sources of information. The responses (Table 3) indicate that social media was the most commonly cited source: 36.2% of all respondents (and fifty-eight percent of those aware of irradiation) reported that they had seen or heard something about food irradiation on social media platforms. Traditional media also played a role: about 27.4% indicated TV or radio as a source of information, and 28.6% cited printed media (newspapers, magazines). Meanwhile, internet websites or news portals were mentioned by 24.8% of respondents, and friends or family by 31.4%. Interestingly, a significant portion (31.9%) said they had heard about food irradiation from official sources or authorities—this could include school/university courses, public health seminars, or information from government agencies. This relatively high figure for authorities likely reflects Kazakhstan’s educational outreach on radiation-related topics due to the Semipalatinsk legacy; some respondents may have encountered discussions on food safety technologies in public forums or through agricultural extension information. In summary, no single information channel dominates, but social media appears to be slightly ahead as the leading source.

These findings highlight that while about half the consumers in the region have at least passing awareness of food irradiation, detailed familiarity is low. It appears that many may have encountered the term in media or conversation, but fewer have a solid grasp of it (as evidenced by the low “very familiar” rate). This sets the stage for examining their actual knowledge and potential misconceptions, which we address next.

### 3.3. Knowledge and Misconceptions About Food Irradiation

To gauge factual knowledge, the survey included true/false statements on key points about food irradiation. Table 4 presents the statements and the distribution of responses (“True”, “False”, or “Don’t know”) for each. The results reveal significant knowledge gaps and misconceptions among the consumers.

Several observations emerge from Table 4. For the statement “Irradiated food becomes radioactive”, only 19.3% of respondents correctly answered “False”. A large majority either thought the statement was true (33.3%) or admitted they did not know (47.4%). This indicates a prevalent misconception: over one-third of consumers mistakenly believe irradiated food is radioactive, and nearly half are unsure. This particular myth is one of the most common globally [9,47,48,49], and our data confirm it is widespread in this region as well. The low percentage recognizing this statement as false suggests a critical knowledge gap regarding the fundamental safety of the irradiation process.

Regarding bacteria reduction, 33.8% answered “True” (which is correct that irradiation kills most bacteria), while 16.9% said “False” and about half did not know. Thus, only one-third of respondents were aware of the primary benefit of irradiation (as a germ-killing technique). The majority either underestimated its efficacy or were uncertain. This is an important educational point: many consumers do not realize that irradiation is a potent tool for eliminating foodborne pathogens [14,50,51].

The statement on recontamination (“Once food is irradiated, it cannot be contaminated again”) was correctly identified as false by just 19.3%, identical to the radioactive statement. Notably, 27.4% believed this false statement to be true, and over half (53.3%) did not know. This suggests some confusion: a segment of people might think irradiation confers some kind of permanent “shield” against microbes, which is not the case. Food can indeed be re-contaminated if mishandled after irradiation (just as pasteurized milk can be contaminated if exposed again). The finding that only one in five know this reflects that even those who may favor irradiation might overestimate its powers, while others simply lack information.

On the topic of nutritional impact, only 20.2% correctly answered that significant nutrient loss is false. About 29.3% thought “irradiation significantly reduces nutritional value” is true, and half did not know. This indicates another common concern: many consumers suspect that irradiated foods might be nutritionally inferior. In reality, most nutritional changes from the low doses used in food irradiation are minor—for example, sensitive vitamins like B1 (thiamine) may be slightly reduced, but overall nutritional content remains largely unchanged, similar to effects of cooking or freezing. Here, nearly 80% of respondents either erroneously believe in large nutrient losses or are unsure, pointing to a potential area for public education (i.e., clarifying that irradiated food retains its nutritional quality).

Finally, for the statement about legal status, 30.2% answered “True” (correct, as Kazakhstan’s food safety law and Codex standards permit irradiation within set limits), 19.5% said “False”, and about half did not know. Therefore, only about one-third of consumers are aware that irradiated foods could be legally sold. The rest either assume it is not allowed or are unsure. This is understandable given that irradiated foods are not yet commonly labeled or found in local markets—consumers have not encountered them, so many might guess they are not permitted. The fact that one in five incorrectly believe irradiated foods are not legal suggests that legal approval alone (as exists) does not translate to public knowledge; outreach would be needed if these products enter the market.

In summary, the overall knowledge level was quite low. If we define a “knowledge score” as the number of statements (out of 5) a respondent got correct, the average score was only about 1.3. Indeed, 91 individuals (21.7%) did not answer a single knowledge question correctly, and another 187 (44.5%) got only one correct. Only five people (1.2%) answered four out of five correctly, and none got all five correct. The most widely known fact was that irradiation can kill bacteria (34% knew this), and the least known (or most misunderstood) were the facts about radioactivity, recontamination, and nutrition (only ~19–20% knew the correct answers for those). These results underscore a clear information deficit: most consumers in the Abai region do not possess accurate knowledge about food irradiation, and many hold incorrect beliefs.

One particularly important insight is that the radioactivity myth persists strongly. This is a critical barrier to acceptance—if a third of consumers actively think irradiated food is radioactive (and nearly half are not sure it is not), any effort to introduce such foods commercially must address this head-on. The data also highlight that “Don’t know” was a very common response (on average, about half of the responses for each item were “Don’t know”), indicating uncertainty. This suggests that many people have not formed a firm opinion on these aspects—which could be positive, in that their views might be swayed by accurate information if provided.

These knowledge findings provide essential context for interpreting the attitude and acceptance results that follow. We next examine how consumers perceive risks and whether they say they would purchase irradiated foods, keeping in mind that many are starting from a point of limited understanding.

### 3.4. Risk Perceptions and Willingness to Purchase Irradiated Foods

The survey probed consumers’ perceived risk of irradiated foods and their willingness to buy or consume such foods. We asked about specific food categories to see if perceptions vary by food type. The results for four key categories (meat, poultry, vegetables/fruits, and baby food) are presented in Table 4, which juxtaposes the perceived risk and willingness responses.

Perceived Health Risk: A majority of respondents expressed some level of concern that consuming irradiated food could be harmful. For each category, over half of the respondents answered that it was “Likely” that eating irradiated versions of that food would harm their health. This was highest for infant food: 60.0% believed irradiated baby food would likely be harmful. For irradiated vegetables or fruits, 57.6% said “likely harmful”, similarly 56–57% for meat and poultry. In contrast, only about 17–23% (depending on food type) thought it was “Unlikely” to be harmful. The remaining ~20–23% were neutral or unsure (Table 4). These figures indicate a generally high level of perceived risk. In other words, roughly three in five consumers assume irradiated foods pose a health risk, which is a substantial hurdle for acceptance. Baby food triggered the highest concern (only 16.9% said unlikely harmful, the lowest confidence of safety among the categories), possibly reflecting extra caution when it comes to infant nutrition.

Willingness to Consume/Purchase: Despite the above risk concerns, the expressed willingness to buy or try irradiated foods was moderate to fairly high. About half of the respondents said they would likely buy/consume irradiated meat (52.6%), vegetables (49.5%), or poultry (48.6%). For baby food, 54.3% said they would be likely to use it if irradiated. The proportions who said they would be unlikely to buy were smaller: 17–21% across those categories (lowest being baby food at 16.9% unwilling, and highest being vegetables at 21.0% unwilling). The rest were neutral (about 28–31% depending on item). These results might seem somewhat contradictory to the risk perceptions—one might expect that those perceiving high risk would not be willing to consume. However, here we see about half indicating willingness, even though a similar or larger fraction sees risk. This apparent paradox is discussed later, but it suggests that many consumers are open to the idea of irradiated food in principle, or at least they are not outright rejecting it, despite harboring safety concerns. It may be that some respondents interpret “willing to buy” as meaning “willing to try it out” or they trust that if it is on the market it might be okay. Alternatively, some could be considering potential benefits (like longer shelf life or fewer pesticides) alongside the risks.

From Table 5, we see that across all food types, a majority perceive irradiation as likely to pose some harm, yet roughly half are still inclined to consider buying. The gap between the “likely harmful” percentages and “unlikely to buy” percentages is notable. For instance, in vegetables, 57.6% say likely harmful, but only 21.0% say they would avoid buying—implying a significant number of people (including some who think there is a risk) still might buy irradiated vegetables. This might indicate a risk-benefit calculus: consumers might be thinking, “Yes, it might be somewhat risky, but I might still buy it if it has benefits or if I trust it enough”. It could also reflect uncertainty (some who said “likely harmful” might not be completely against trying it, especially if neutral on buying). Another interpretation is that these questions tap into slightly different psychological constructs: one is a perceived hazard, the other is a behavioral intention that could be influenced by factors beyond hazard perception (such as trust in regulation or perceived benefits).

We further examined whether consumers differentiate much between food categories in terms of risk or willingness. Overall, differences between food types were modest. A Friedman test found no statistically significant difference in the distribution of risk perceptions among meat, poultry, vegetables, and baby food (*p* = 0.44), confirming that risk was generally viewed as high for all. For willingness, similarly, no significant differences were found across those categories (*p* = 0.43)—willingness rates hovered around 50% for each. One trend worth noting is the slightly higher concern for infant food: “unlikely harmful” was lowest for baby food at 16.9%, and conversely, “unlikely to buy” was also lowest for baby food at 16.9%. This suggests that while more people fear irradiated baby food could be harmful, a slightly higher proportion are still willing to use it (perhaps those who trust that if it is approved for babies, it must be safe). This could be influenced by the sample’s age distribution—many older respondents (grandparents) might say it is harmful and they would not use it, whereas younger respondents (some without kids) might answer more hypothetically. Indeed, we found an age interaction (discussed below): older adults were more likely than younger ones to say “unlikely to buy” baby food (see Factors Influencing Acceptance).

**Figure 3:**
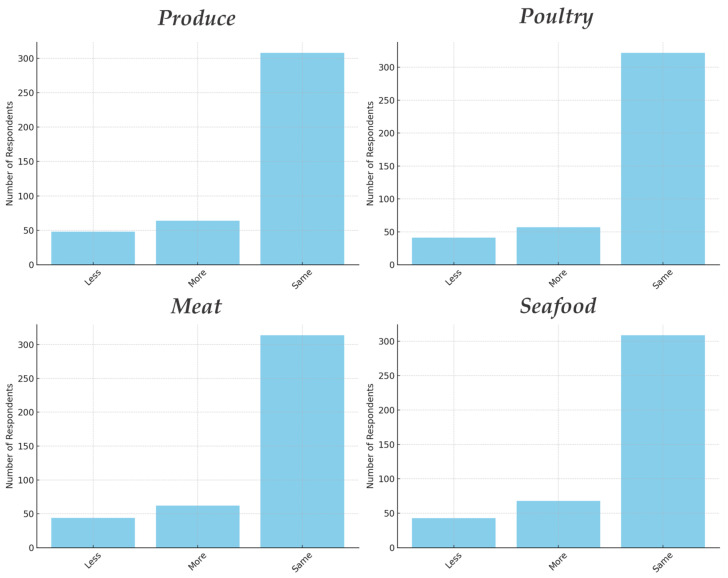
Purchase intentions bar charts showing if respondents would buy more, the same, or less than currently for fresh produce, poultry, red meat, seafood.

To get a sense of how irradiation might affect consumer behavior overall, we also looked at the purchase intent question (more/same/less). As seen in Figure 3 the majority of respondents (around 73–77%) said they would buy “about the same amount” of their usual foods if those foods were irradiated (indicating no change in consumption). Meanwhile, those who said they would buy more outnumbered those who said less for every category. Specifically, about 13–16% of respondents said they would buy more irradiated produce, meat, poultry, or seafood than currently, whereas only about 9–11% said they would buy less. This yields a net positive inclination. For example, for seafood, 16.2% would buy more if it were irradiated (perhaps due to perceived safety from parasites or longer shelf-life), versus 10.2% who would buy less (likely due to fear), with 73.6% no change. These findings align with the willingness rates—they suggest that the introduction of irradiated foods might not drastically reduce consumption and could even increase it slightly for some consumers, assuming price and other factors constant. The majority are indifferent (no change), which often implies acceptance of the status quo; a smaller group is enthusiastic (more), and an even smaller group is avoidant (less).

To summarize the risk and willingness results, public perception in the Abai region currently errs on the side of caution—most believe irradiated foods could be risky. However, in a seemingly paradoxical way, many people are still open to trying or buying irradiated foods. This indicates that initial acceptance might be reasonably good (with about half the market willing to give it a chance), but underlying safety worries need to be addressed to convert that tentative acceptance into sustained confidence. Our data suggest an opportunity: since a large fraction of people are not outright rejecting irradiated foods, effective communication and education could tip many of the “neutral” or even some “likely harmful but maybe I’ll try” individuals into comfortable consumers, especially if they see tangible benefits.

### 3.5. Factors Influencing Acceptance and Attitudes

We investigated whether certain subgroups of consumers differed in their perceptions or acceptance of irradiated foods, and whether correlations exist between knowledge/attitudes and willingness. Below we outline significant findings (or notable non-findings) regarding the influence of knowledge, trust, age, education, and other factors.

Knowledge vs. Attitudes: One might hypothesize that people who know more facts about irradiation (for instance, those who correctly dismiss myths) would be more accepting and perceive lower risk. However, in this sample, we did not find strong linear correlations between the knowledge score (0–5) and either risk perception or willingness. Even those with above-average knowledge still expressed considerable concern, and conversely, even those with misconceptions sometimes indicated willingness to buy. For example, among respondents who mistakenly believed “irradiated food becomes radioactive”, over half still said they would likely buy irradiated food (whereas one might expect them to avoid it). This apparent disconnect suggests that other factors (perhaps general attitudes or trust) might be moderating the relationship between knowledge and acceptance. It could also indicate that the overall knowledge level is so low that differences within the range (0, 1, or 2 correct answers) are not enough to shift attitudes substantially—essentially, most people, even the “knowledgeable”, still had incomplete understanding, so all groups had reservations.

One interesting nuance: those few respondents who knew the food would not become radioactive were not significantly more willing overall than those who did not know this. In fact, their willingness rates were quite similar (and in some categories slightly lower, though sample size is small). This might reflect a cautious outlook even among the informed—possibly because those who took the effort to learn about irradiation also learned about potential downsides, or because knowledge came from academic/scientific backgrounds that still approach new food tech carefully. It is also possible that the causality is reversed: individuals who are more skeptical or concerned might have sought more information (thus answering knowledge questions right) but remain skeptical. In any case, the data indicate that simply having factual knowledge is not a guarantee of acceptance; risk perception and acceptance seem to depend on more than factual understanding alone, which is consistent with the literature on risk psychology where emotions and trust play a role [2,9,44,45,46,47].

Education: Education level showed some significant associations with certain perceptions. Notably, education was related to the perceived necessity of irradiation for certain foods. Chi-square analysis revealed that respondents’ education level significantly affected their ratings of necessity for fruits (*p* = 0.047) and especially for vegetables (*p* = 0.010). In particular, those with intermediate education (some college/vocational) or high-school education were more likely to say irradiating fruits and vegetables is “Very necessary”, compared to those with a university degree. For instance, 27.3% of high-school-educated respondents and 28.6% of vocational-trained said irradiation is very necessary for vegetables, while only 11.9% of those with a bachelor’s degree did so (most of the latter chose “somewhat” or “not necessary”). A one-way ANOVA on the numeric necessity score for vegetables by education was significant (*p* = 0.027), and Duncan’s post hoc test indicated that the bachelor’s+ group had a significantly lower mean necessity rating than the other groups, who were higher (the latter two groups did not differ from each other significantly). A similar (though slightly weaker) trend was seen for fruits: the bachelor’s+ group had the lowest proportion saying “very necessary” (~16%), whereas the some-college group was highest (~28.6% saying very necessary for fruit). This pattern could imply that the most educated consumers are actually more skeptical about the need for irradiation on produce—they may believe other methods (proper washing, traditional preservation) suffice, or they might be more aware of debates around irradiation. Conversely, those with some college or technical education might have learned about issues like food spoilage or contamination in a practical context and thus appreciate irradiation’s benefits for produce. It is also possible that higher-educated individuals are more likely to have an initial cautious stance pending more evidence, whereas mid-educated individuals trust what they have heard about benefits. This is speculative, but the data clearly indicate an education-related difference in perceived need for the technology, especially in the context of produce.

**Figure 4:**
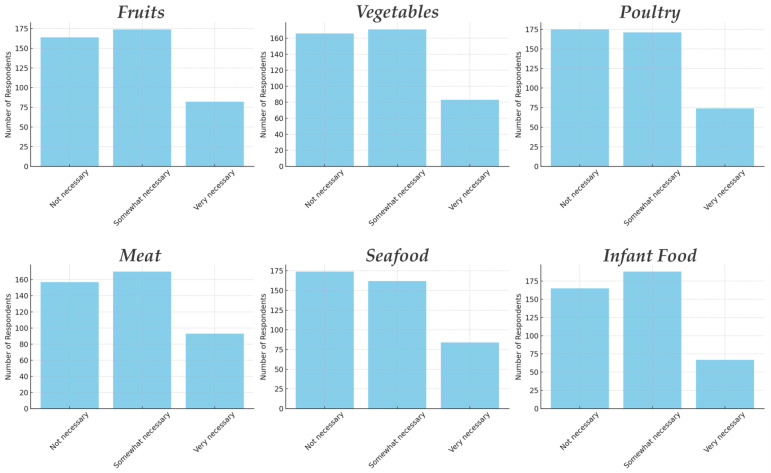
Consumer perception of how necessary irradiation is for different food types.

Despite these differences in perceived necessity, education did not show a clear effect on willingness to buy or on perceived risk. All education groups had similarly high risk perceptions and around 50% willingness rates. For example, willingness to buy irradiated meat was ~51% for bachelor’s, ~52% for high school, ~55% for some college (no significant difference). This suggests that while education influences how “necessary” people think irradiation is in theory, it has not yet translated to differences in their stated behavior or risk views. Perhaps even those who think it is very necessary still have concerns, and those who think it is not necessary might still consider buying it if offered. The mixed influence of education underscores that acceptance is not a straightforward function of formal education level. Responses on the necessity of irradiation on different groups of food products can be seen in Figure 4.

**Figure 5:**
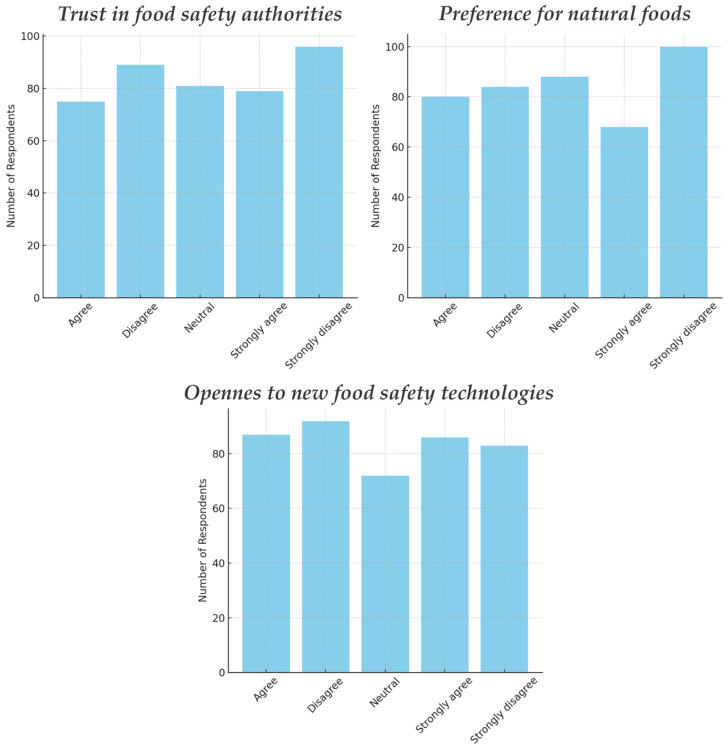
Charts reflecting consumers trust in food safety authorities, their preference for natural foods and their openness to new food safety technologies.

Trust in Authorities: Trust in regulatory bodies and scientific authorities could logically influence willingness—if one trusts that authorities ensure safety, one might be more willing to accept an irradiated product. As seen in Figure 5, in our survey, trust levels were fairly mixed (only about 18% strongly agreed they trust authorities on this, 17% agreed, 19% were neutral, and 46% disagreed to some degree). When splitting respondents by high trust vs. low trust, their willingness rates for various foods were not significantly different (chi-square tests *p* > 0.5). This was somewhat surprising. It may be that those who trust authorities already assume that if a food is allowed it is fine—but simultaneously those who distrust authorities might still consider buying if they judge the product on other merits. Another possibility is that many respondents answered the trust question generally (their general trust stance) but when considering actual purchase, they factored in more concrete considerations (like perceived benefit of the product). The lack of correlation could also be due to the polarized but balanced trust responses—with nearly half low trust and half neutral/high, their behavior might average out similarly.

However, we did observe a very slight negative correlation between familiarity with irradiation and trust in authorities (r = −0.096, *p* = 0.048, see Table 5 later). This suggests that those who claimed higher familiarity tended to be less trusting of authorities on this matter. While the correlation is weak (just at significance), it hints that individuals who have actively learned about irradiation (perhaps through informal sources or personal research) might rely less on government assurances and more on their own judgment—potentially because they have encountered conflicting information or simply have a more questioning attitude.

Overall, no single psychological factor we measured (knowledge, trust, naturalness orientation, tech optimism shown in Figure 5) showed a dominant effect on acceptance. This suggests that consumer attitudes toward irradiated food might not be deeply rooted yet; many people are essentially undecided or ambivalent, influenced by a mix of fragmentary knowledge and general food safety concerns. This ambivalence is actually an opportunity: research elsewhere has shown that providing balanced, clear information can shift consumer attitudes significantly [13,16,17,52]. The fact that we did not see strong polarization means many could be swayed toward acceptance with the right approach.

Gender: There were no significant gender differences in familiarity, knowledge, risk perception, or willingness to buy irradiated foods. Men and women in our sample had very similar response distributions (Table 5 includes correlation and chi-square analyses). For instance, 49.5% of males vs. 55.6% of females said they would likely buy irradiated meat (not a significant gap), and 56.8% of males vs. 51.9% of females for baby food, etc. Chi-square tests for willingness by gender were *p* > 0.1 in all cases, indicating no evidence of a true gender effect. This suggests that both men and women share comparable levels of concern and openness; any stereotype that perhaps women (who often handle food prep) might be more wary, or that men might be more technology-positive, was not borne out here.

Age: When treating age as a continuous variable, there was no notable correlation with overall willingness (r ≈ −0.066, *p* = 0.18) or risk perception (r ≈ −0.016, *p* = 0.74). However, when we binned age into groups, an interesting pattern emerged specifically for infant food acceptance. The older group (60+ years) was significantly more likely to say they would not use irradiated baby food compared to younger groups (chi-square *p* = 0.048). In the oldest group, 22.9% said they were “Unlikely” to buy irradiated infant food, versus only 10.1% of the 18–29 group (with the 30–59 group at ~15.4%). Younger adults (under 30) actually had the highest “likely to buy baby food” rate (55.7%) and lowest concern, while seniors had the lowest “neutral” and a higher split between likely and unlikely. This likely reflects that older respondents, who may be answering from the perspective of protecting grandchildren or drawing on traditional views, exhibit more caution for the most vulnerable (infants). Younger respondents, many of whom might not have children, could be less emotionally invested or simply more trusting in that scenario. Outside of the baby food context, age did not show significant differences in acceptance of other foods. We did not find evidence that the very young or very old were systematically more pro- or anti-irradiation overall. Thus, apart from a specific older-generation caution toward infant food, age differences were minor. This is somewhat intriguing because sometimes younger consumers are thought to be more accepting of new food technologies (being more accustomed to them), but here even the 18–29 group had nearly the same willingness percentages as other adults for general foods. They did, however, show less apprehension for baby food, which might simply be due to not imagining feeding a baby themselves yet.

Urban vs. Rural, and Location: We checked if living in an urban environment (with presumably more exposure to technology and information) versus a rural one affected responses. There were no large differences: familiarity was only slightly higher in urban respondents (more had heard of it, but not statistically significant), and willingness and risk perception were similar. One minor note: rural respondents were somewhat more likely to accept irradiated vegetables—56% of rural folks said likely to buy vs. 45.5% of urban, and only 16.7% rural vs. 23.5% urban said unlikely to buy vegetables (*p*~0.08). This was not statistically significant at 5% but hints that rural people—who might worry about pests or spoilage of produce—could see irradiation as beneficial there. Regarding the five specific locations sampled, we did not find major differences in attitudes. Semey city respondents showed slightly higher trust in authorities and a somewhat higher fraction viewing irradiation as safe (33% in Semey said unlikely harmful for vegetables, vs. only ~12% in one rural district, Zharma), but these differences were not statistically significant overall (chi-square *p* > 0.1). The consistency across locations might be partly because overall knowledge was uniformly low, and perhaps regional culture in Abai is relatively homogenous on this topic. The town of Kurchatov (near the nuclear test site) did not show a markedly different pattern of responses compared to others; one might have hypothesized either greater acceptance (due to scientific community presence) or greater fear, but our data for Kurchatov (though a smaller subsample, n = 62) did not stand out significantly in either direction.

To complement these subgroup analyses, we constructed a Pearson correlation matrix (Table 6) for key continuous/ordinal variables: age, familiarity level, knowledge score, perceived risk index, willingness index, and the attitude scales (trust, naturalness preference, tech acceptance). This gives an overview of linear relationships. As noted, most correlations are close to zero, reflecting the generally weak associations. The only statistically significant correlations was a negative correlation between trust in authorities and familiarity (r = −0.10, *p* ~ 0.048).

The matrix confirms that no strong linear relationships exist between these factors in the data, demographic factors and general attitudes only explain a small amount of variation in perceptions at this stage. The broad trend is that perceptions of irradiation were consistently cautious across different groups, with only a few differences, older people more protective of baby food, highly-educated slightly more skeptical of need, etc. This uniformity might suggest that the concept of food irradiation has not been politicized or polarized among the public; rather, most people share a common baseline of low information and moderate concern. This is arguably a favorable situation for intervention: with effective public education campaigns tailored to highlight the safety and benefits of irradiation, for example, emphasizing that it can prevent diseases like intestinal infections without compromising food quality, there is potential to raise acceptance significantly. Past studies have shown that when consumers are educated about irradiation in clear terms and even given a chance to see/taste irradiated products, acceptance jumps appreciably [17].

Our findings also imply that addressing key misconceptions should be a priority. If consumers come to understand that irradiated food is not radioactive and remains wholesome, their risk perceptions may moderate. Additionally, the fact that willingness to try is already around 50% is encouraging—it means half the population is already open-minded enough to consider irradiated foods. The remaining neutrals and even some of the initially unlikely could be converted by trust-building measures, such as endorsements by health professionals or seeing others adopt it safely.

Finally, it is worth noting that, given the historical context of the region, we did not find evidence of an exaggerated fear of anything “radiation”-related—concerns were high but within the range seen in other countries lacking public education on the topic. People did not outright reject irradiation en masse, only ~10–20% said they would avoid irradiated foods. This suggests that the legacy of Semipalatinsk, while making radiation emotive, has not made the public completely averse to radiation technologies in all forms. Indeed, Kazakhstan operates radiation facilities and the public seems to distinguish nuclear weapons radiation from controlled beneficial uses to some extent. This nuance is important: it means discourse can be framed positively, focusing on how irradiation is a solution for safer food, analogous to pasteurization, often termed “cold pasteurization” in literature [6,53], rather than invoking nuclear imagery.

## 4. Discussion

This study provides one of the first data-driven insights into Kazakhstani consumer perceptions of food irradiation, using a real sample from the Abai region. The findings highlight a classic paradox often seen with novel food technologies: widespread lack of knowledge and presence of misconceptions, paired with a tentative openness to try the products. In the Abai region, consumer awareness of food irradiation is moderate—about half have heard of it—but detailed understanding is poor, as evidenced by the low quiz scores and pervasive “don’t know” responses. The majority of consumers hold cautious attitudes, considering irradiated food risky, yet about half are still willing to consider purchasing such foods.

One of the most salient results is the confirmation that the radioactivity myth is alive and well among consumers here. Nearly half of respondents did not know that irradiated food is not radioactive, and one-third actively believed it becomes radioactive. This misconception has been documented in many other settings—for example, Resurrección et al. (1995) found “over 30% of consumers believe that irradiated food is radioactive” [9] in an American sample, and more recent studies continue to report similar figures worldwide [1,12,13,16,49]. Our data, with ~33% believing it and ~47% unsure, suggest an even larger uncertainty around this point in Abai Region, Kazakhstan. Clearly, any consumer education initiative should tackle this fundamental misunderstanding. Emphasizing the physics (that the energy is not retained, analogous to an X-ray scan not making your body radioactive) in layman’s terms will be crucial. It may be useful to draw analogies to pasteurization or cooking: the food is exposed to something that kills germs, but the food itself does not become the source of anything dangerous afterward.

Correspondingly, the perceived risk of irradiated foods was high in our study. Around 56–60% thought health harm was likely from eating irradiated items. This aligns with a general negative perception observed in many countries when people first learn about irradiation. For instance, some European consumer studies have shown initial high risk perceptions that drop after information is provided. It is notable, however, that consumers in our sample rated irradiation’s risk roughly on par with each other across different foods—they did not single out, say, meat as more dangerous than vegetables. In contrast, in some contexts consumers worry more about irradiated meat (perhaps associating it with radiation in flesh) or worry about specific categories like baby food. We saw slightly elevated concern for baby food, which is understandable given the sensitive population, but overall, risk concerns were broad. This broad-brush concern likely stems from the general notion of “radiation = bad” without finer distinctions. It suggests that once the general fear is addressed, it could alleviate concerns across the board.

Despite high risk perceptions, about half of the respondents indicated willingness to buy irradiated foods. This finding is consistent with prior studies that often find a substantial minority to majority of consumers are willing to try irradiated foods when asked hypothetically. For example, in the 1995 Georgia survey, 45% said they would buy irradiated food and 19% would not [9], very close to our meat willingness results (53% yes, 17% no). More recently, the global systematic review reported that as of 2024, about 67% of consumers globally accept irradiated food [15]. Our figure ~50% “likely to buy” is a bit lower than the global average, which is expected as Kazakhstan (a developing country) tends to have slightly lower acceptance compared to highly industrialized countries. It is plausible that with more exposure and education, Kazakhstan’s acceptance levels could approach the global 67% benchmark. Additionally, our scenario was hypothetical; actual behavior might differ, but studies show that stated willingness often translates reasonably well into trial behavior, especially if products are properly introduced [19,54].

The simultaneous existence of high risk concern and moderate willingness might seem contradictory, but it reflects the complex nature of consumer decision-making. Several factors could explain it. First, some respondents might lack conviction in their risk assessment—they say “likely harmful” because intuitively it sounds risky, yet they are not entirely sure and would still try it if others do or if convinced otherwise, as was observed by Behrens et al. and Tatum et al. [12,55]. Second, consumers often balance perceived risks against perceived benefits (a risk–benefit trade-off). As observed by Ablan et al. [11], Parrella et al. [56] and van Dijk [57]. It is possible that in their minds, irradiation has some benefits (perhaps longer shelf life, fewer insects or chemicals, improved food safety) that somewhat offset the risks, making them at least open to trying it once. Third, trust in the system might implicitly be at work: a consumer might personally feel uneasy but think “if the authorities allow this, maybe it’s okay—I might try it cautiously”. The “recreancy theorem” explains that when people lack detailed knowledge, they may still accept a technology if they trust that authorities are managing it safely [58,59]. The context of Kazakhstan is relevant here: the government has actively promoted nuclear technology for peaceful uses (e.g., recent referendum on nuclear power plant construction, proposal carried with 73.11% approval) but the public remains wary. In the case of food, there has not been a national campaign on irradiation, so people have not formed strong opinions yet. This latent openness combined with caution is actually a typical starting point observed for other food technologies historically (for example, pasteurization of milk in the early 20th century had its skeptics initially, or GMOs in the 1990s [60]—though GMOs eventually faced more entrenched opposition due to ethical/naturalness debates, which irradiation largely avoids [10]).

In terms of psychological theory, this scenario can be partly interpreted through Protection Motivation Theory (PMT). According to PMT, people faced with a potential threat (here, the perceived risk of irradiated food) will decide on protective action based on both threat appraisal (how severe and likely the threat is) and coping appraisal (how effective and feasible is the protective response). In our case, many perceive a threat (health risk), but their coping response (avoiding irradiated food) may not be strongly activated because: (a) they also perceive potential benefits or necessity (reducing another threat like food poisoning), and (b) they have some trust that authorities would not allow an extreme danger (response efficacy of regulation). Thus, rather than outright rejecting the product to “protect” themselves, many remain on the fence or cautiously willing to try it, especially if provided with reassurance. This risk–benefit balancing act, where consumers weigh the potential safety improvements against their fear of the technology, explains why high concern can coexist with moderate openness. This interpretation is supported by recent findings that show perceived benefits and response efficacy play significant roles in motivating consumer behavior toward food safety decisions, even when threat perception is high [56,61]. Studies applying PMT to food-related threats show that while perceived severity and vulnerability contribute to threat appraisal, coping appraisal (particularly self-efficacy and belief in regulatory systems) more strongly influences consumer openness to new technologies [62,63]. Another important finding is that socio-demographic differences are not pronounced for these attitudes. This means the acceptance of irradiated food is fairly broad-based and not confined to specific subgroups, which is encouraging for any broad public health messaging. However, the differences we did find offer insight into targeted strategies: socio-demographic differences are not pronounced for these attitudes. Acceptance of irradiated food is fairly broad-based and not confined to specific subgroups, which is encouraging for any wide-reaching public health intervention. However, the subtle differences we did find offer insight into targeted strategies. The fact that older adults are more hesitant about infant food suggests that special attention should be given to messaging for products like baby food if irradiation were used there. Grandparent-age individuals might need reassurance from pediatricians or health organizations emphasizing that irradiated baby food is safe—perhaps even safer than non-irradiated, since it would have no pathogens. Likewise, the education-level difference in perceived necessity indicates that outreach might need to be nuanced. Highly educated consumers might respond better to data-driven communication (they might want to see evidence of need, e.g., statistics on foodborne illness rates or post-harvest losses that irradiation could reduce). Meanwhile, less-educated consumers who already see irradiation as necessary might just need reassurance and practical information (they may be sold on the idea that it is useful, but just want to know it is safe). It is interesting that those with some technical education were quite supportive of the necessity—this group could actually become opinion leaders if engaged properly, as they might be respected in their communities for having some technical know-how.

In terms of general attitudes, one might have expected that people who strongly prefer “natural” foods would oppose irradiation (viewing it as an unnatural process). Our data did not show a strong link; perhaps because even natural-food lovers lack knowledge about irradiation, they have not firmly decided if it is “unnatural” like they might view GMOs or additives. This aligns with findings that many consumers, even when aware of food irradiation, report low understanding and often confuse it with radioactivity, which leads to hesitation rather than firm opposition [9,12]. Similarly, trust in authorities was not high on average but also not strongly tied to acceptance. This could mean that people are relying on their own gut feelings rather than what authorities say, which again underscores the need for informative and persuasive communication. Research confirms that public trust in institutions does not consistently predict acceptance of food irradiation, with consumer decisions often guided more by personal heuristics and emotional reactions than by institutional endorsements [56,59]. The lack of gender difference simplifies outreach—messages do not need to be gender-targeted; both male and female shoppers have similar concerns.

Urban vs. rural parity suggests that even rural populations (who sometimes are harder to reach with sophisticated info) are not particularly more fearful; if anything, some rural folks might welcome it for produce preservation. This means rural extension programs could integrate education on irradiation alongside other food safety training without expecting huge cultural resistance.

One surprising result was that general trust in authorities did not map clearly to acceptance. In many cases, trust is a predictor of accepting scientific assurances. The absence of a strong link here could mean that even those who trust the government have not heard the government say much on this issue yet—so their trust has not been called into action. Meanwhile, those who distrust might still be willing to try if they see personal benefit. It underscores that trust, while important, may not be the decisive factor at this stage; knowledge and clear information might matter more in forming initial opinions. That said, building trust will be important going forward. As authorities start to speak more on this (if Kazakhstan pursues irradiation use), maintaining transparency will be key. Our data showed a roughly even split between those inclined to trust and those inclined not to—a “neutral” trust stance for many. This is a somewhat malleable situation; consistent messaging and perhaps third-party endorsements (academics, independent consumer organizations) could help assure the skeptics.

It is worth noting that, given the historical context of the region, we did not find evidence of an exaggerated fear of anything “radiation”-related beyond what is observed elsewhere. Concerns were high but within the range seen in other countries that lack public education on the topic. People did not outright reject irradiation en masse; only about 10–20% said they would avoid irradiated foods entirely. This suggests that the legacy of Semipalatinsk, while making “radiation” an emotionally charged term, has not made the public completely averse to radiation technologies in all forms. Indeed, Kazakhstan operates radiation facilities (for research, sterilization, etc.), and the public seems to distinguish nuclear weapons/radioactive fallout from controlled, beneficial uses to some extent. This nuance is important: it means the public discourse can be framed positively, focusing on how irradiation is a solution for safer food (often dubbed “cold pasteurization” in literature [6,53]) rather than invoking nuclear imagery.

### 4.1. Practical Implications

In moving forward, practical implications can be drawn from our findings. First and foremost, targeted education programs are needed to explain what food irradiation is and is not. These should debunk the key myths (highlighting that irradiated foods are not radioactive and remain nutritious) and communicate the proven benefits for food safety and longevity. Such education could be integrated into public health messaging or even school science curricula; indeed, other countries have piloted school-based programs to teach students about irradiation, yielding improved understanding and acceptance as has been done in Korea and reported by Cho et al. [64]. Second, it will be important to leverage trusted sources and relatable examples in outreach. Endorsements from medical professionals, food safety experts, or even testimonials from countries where irradiated foods have been consumed safely could help alleviate fear. Given Kazakhstan’s nuclear history, messaging might need to acknowledge the past testing and clearly distinguish it from controlled food irradiation to preempt confusion. Third, transparent labeling and information at the point of purchase will be crucial. Introducing the internationally recognized Radura symbol (the logo indicating an irradiated product) alongside plain-language explanations can ensure consumers feel informed rather than deceived. Many jurisdictions worldwide require such labeling [65], and doing the same in Kazakhstan with proper public awareness can build trust. It should be emphasized that irradiation is a complementary safety measure akin to pasteurization—not a replacement for overall quality but an added layer of protection that has public health benefits. Fourth, framing the consumer benefits is key to a positive narrative. Messaging can highlight immediate advantages that matter to consumers: safer food (less chance of food poisoning), longer shelf life (e.g., fruits that stay fresh longer without rot, grains without insect infestation), and potentially reduced need for chemical preservatives or fumigants. If consumers see a direct benefit to themselves or their family, they are more likely to view the technology favorably. For example, educated consumers might appreciate knowing that spices can be sterilized by irradiation instead of using chemicals, resulting in a purer product. Finally, addressing specific concerns of subgroups can fine-tune the approach. For older generations and sensitive products like baby food, extra reassurance from pediatricians or child health experts might be needed to build confidence. For highly educated or scientifically minded consumers, providing access to the data (e.g., published research, risk assessments) may increase their confidence and even turn some into advocates among their peers.

This research confirms that consumer education is essential and likely effective for improving acceptance of irradiated foods in Kazakhstan. The findings echo global recommendations that information should focus on immediate consumer benefits of irradiation—for instance, emphasizing reductions in food poisoning cases, increased shelf life (meaning fewer grocery trips, economic savings from less spoilage), and possibly reduced need for chemical treatments. Messages should also tackle the top concerns: reassure that the food is not radioactive and that nutritional and sensory qualities remain intact. Transparency about what is known and not known can further build trust [66,67]. Importantly, communication should come from trusted voices and be delivered in accessible language. As noted by previous researchers, “using layman-suitable terminology” and sharing information in a transparent way can mitigate the fear of novel technologies [68,69,70]. In Kazakhstan, leveraging the trust in scientists could help bridge the gap between regulatory assurances and public belief.

Our data also imply that introducing irradiated foods without prior public education could result in a lukewarm reception. If half the consumers are willing to try but doing so with misgivings, any negative rumor or minor incident could quickly erode trust. Therefore, a well-planned introduction strategy is needed—possibly starting with less-sensitive products and gradually expanding once consumers have positive experiences. The fact that many consumers do not differentiate much between categories means that a success, or failure in one category could spill over to perceptions of others. Therefore, initial introductions need to be carefully managed for success in building a positive halo effect.

Lastly connecting these findings to the broader context of Kazakhstan’s food safety and security goals. The country, being a large exporter of grains and other foods, can benefit from irradiation technology. Consumer acceptance domestically is also important because it affects whether local producers will adopt the technology. If producers sense that consumers will avoid irradiated products, they might be reluctant to invest in it. Thus, demonstrating that consumers can accept irradiated foods is crucial for motivating industry uptake. Our study provides encouraging evidence that with proper guidance, consumers are not averse to the idea.

### 4.2. Limitations

This study has several limitations that should be acknowledged when interpreting the findings. 

First, the sample was obtained through convenience sampling in selected locations, which may limit the generalizability of the results. While we included a mix of urban and rural consumers and various districts, the sample is not a perfect random representation of the Abai region or Kazakhstan as a whole. There may be slight biases such as overrepresentation of certain demographic groups (e.g., individuals with higher education might have been more willing to participate in a survey about a technical topic). Consequently, the overall levels of awareness or acceptance observed might differ from the true population values. 

Second, the data rely on self-reported responses to a hypothetical scenario. Social desirability bias could be at play—some respondents might have stated they are “likely to buy” irradiated food because it seemed like the reasonable or modern answer, even if in reality they would act more cautiously. Conversely, some might understate their willingness due to the unfamiliarity of the concept when, in practice, they might follow others or expert recommendations. Without actual market behavior to observe, we must treat stated intentions as indicative but not definitive. 

Third, we did not explicitly examine the effect of price or cost in this study. Consumers’ willingness to purchase irradiated foods could heavily depend on pricing (e.g., if irradiated products are more expensive due to processing costs). Our questionnaire did not introduce a price component or trade-off, so we cannot gauge how price sensitivity might alter acceptance. This is a variable that future research or market tests should consider, as even intrigued consumers might shy away if irradiated products are priced at a premium and they are not convinced of added value. 

Fourth, we focused on perceptions and attitudes at one point in time, shortly after basic exposure via the survey itself. We did not provide an educational intervention in the survey (beyond asking questions), so knowledge remained low; however, the act of asking questions might have caused some respondents to think about the issues in real-time, potentially influencing their later answers (for instance, considering the knowledge quiz before answering willingness could create some ambiguity or reflection that wouldn’t occur in a naive state). 

Fifth, this study did not explicitly assess consumer perceptions of the sensory quality of irradiated foods—such as taste, texture, appearance or aroma. Moderate irradiation (3–6 kGy) may positively influence tenderness with minimal adverse sensory impact [71,72]. However, higher doses (9 kGy or more) may lead to substantial structural damage [73], potentially reducing consumer acceptance due to compromised texture and perceived freshness [74]. These sensory factors, though not explored in our survey, may affect consumer willingness more strongly than abstract risk perceptions. Additionally, the role of product-elicited emotions—such as anxiety, distrust, or disgust associated with radiation-based processing—was not measured. Emotions play a key mediating role between perceived risk and behavioral intentions, as shown in food technology literature [11,12,75]. 

There are other unmeasured factors that could influence acceptance. Personal values or worldviews (beyond the few attitudes we measured)—such as general risk aversion, scientific literacy, or trust in specific institutions—could play a role but were not directly captured. Cultural and historical factors specific to the region (such as personal or family experiences with radiation from Semipalatinsk) were not explicitly probed in the survey and could add nuance to understanding responses. These limitations suggest caution in overgeneralizing results, and point to the need for further studies, perhaps with broader sampling, experimental information provision, and inclusion of economic considerations.

## 5. Conclusions

This research reveals that consumers in the Abai region of Kazakhstan currently have limited knowledge but a cautiously open attitude toward food irradiation. Misconceptions—especially the false belief that irradiated food becomes radioactive—are common and contribute to elevated risk perceptions. Nonetheless, about half of consumers indicate a willingness to purchase irradiated foods, and most others are neutral or undecided, with only a small minority firmly opposed. No strong demographic divides were observed in these attitudes, suggesting that public perception is fairly uniform in its baseline state of uncertainty and mild concern. This uniformity provides an opportunity: a well-designed public education campaign can broadly raise awareness and understanding, thereby shifting consumer attitudes in favor of irradiated foods. Continued research and monitoring are advised. As educational efforts are implemented and if irradiated products enter the market, follow-up studies should measure changes in consumer knowledge and acceptance. Tracking these trends will help ensure that scientific progress in food safety is matched by public confidence, resulting in the successful integration of the technology for the benefit of all.

The results of this study imply that Kazakhstan stands at a pivotal point regarding consumer acceptance of food irradiation. Public sentiment is not entrenched against it; rather, it is malleable and likely to improve with knowledge and positive exposure. Learning from international experiences, it is evident that consumer acceptance tends to grow over time as people become more familiar with irradiated products and realize they have consumed them without issue. In the global context, with roughly two-thirds of consumers now reportedly accepting irradiated food, Kazakhstan’s consumers can similarly move in that direction through effective engagement and education.

Our study underscores the importance of bridging the current “awareness gap”: turning that ~50% who have only vaguely heard of irradiation into a well-informed majority who understand its purpose and safety. By doing so, Kazakhstan can harness the full benefits of food irradiation technology—such as a safer food supply, reduced post-harvest losses, and improved public health—while maintaining consumer trust and satisfaction. The consumer is a key stakeholder in the food safety equation, and our findings provide a baseline for incorporating consumer perspectives into the rollout of irradiation as a food safety intervention in Kazakhstan and similar settings.

## Figures and Tables

**Table 1:** Overview of the questionnaire structure and its contents.

Section	Content and Example Items	Response Format
Demographics	Age, gender, ethnicity, education level, monthly income, area of residence (urban/rural)	Various (age in years; multiple-choice for others)
Familiarity	Self-rated familiarity with food irradiation (e.g., “How familiar are you with food irradiation?”)	5-point scale—“Not at all” to “Extremely” familiar)
Information Sources	Whether the respondent has heard about food irradiation from specific sources (TV/radio, Internet news, social media, friends/family, printed media, authorities/education)	Yes/No for each of 6 sources
Knowledge	Five true/false statements about irradiation (e.g., “Irradiated food becomes radioactive”—correct answer False)	“True”, “False”, or “Don’t know” for each statement
Risk Perception	Perceived health risk if certain foods were irradiated (red meat, poultry, fruits/vegetables, infant/baby food)	3-point scale “Likely” harmful, “Not sure/Neutral”, “Unlikely” “Harmful” for each food category
Willingness to Consume	Willingness to eat or purchase those irradiated foods (red meat, poultry, fruits/vegetables, infant food)	3-point scale (“Likely”, “Neutral”, “Unlikely” to consume) for each category
Purchase Intent	If foods were irradiated for safety, whether the respondent would buy more, the same, or less than currently for: fresh produce, poultry, red meat, seafood	3 options (“Buy more”, “About the same”, “Buy less”) for each food group
Perceived Necessity	Perception of how necessary irradiation is for different food types (fruits, vegetables, poultry, red meat, seafood, infant food)	3-point scale (“Not necessary”, “Somewhat necessary”, “Very necessary”) for each category
General Attitudes	Attitudinal statements influencing acceptance: (1) Trust in authorities to ensure irradiated food is safe; (2) Preference for natural (unprocessed) foods; (3) Openness to new food technologies for safety	5-point Likert agreement scale (1 = Strongly disagree, 5 = Strongly agree) for each statement

**Table 2:** Demographic characteristics of survey respondents (N = 420). Values are number of respondents and percentage in parentheses.

Characteristic	Category	n (%)	Kazakhstan, c. 2021 (%)
Population	All	420 (100)	19,186,015 (100)
Gender	Male	206 (49.0)	9,324,840 (48.6)
	Female	214 (51.0)	9,861,175 (51.2)
Age group	18–29 years	79 (18.8)	2,674,322 (23.2)
	30–59 years	201 (47.9)	7,315,163 (63.5)
	≥60 years	140 (33.3)	1,526,460 (13.2)
Ethnicity	Kazakh	283 (67.4)	13,497,891 (70.4)
	Russian	114 (27.1)	2,981,946 (15.5)
	Other	23 (5.5)	2,706,178 (14.1)
Education	High school or less	139 (33.1)	5,400,586 (39.95)
	Some college/Vocational	105 (25.0)	4,386,963 (32.45)
	Bachelor’s or higher	176 (41.9)	3,731,201 (27.6)
Monthly Income	<100,000 KZT	83 (19.8)	9,750,362 (63.33)
	100–199,000 KZT	120 (28.6)	3,908,541 (25.38)
	200–299,000 KZT	116 (27.6)	1,532,055 (9.95)
	≥300,000 KZT	101 (24.0)	206,667 (1.34)
Area of Residence	Urban (cities)	264 (62.9)	11,741,342 (61.2)
	Rural (villages)	156 (37.1)	7,444,673 (38.8)
Survey Location	Semey (city)	91 (21.7)	
	Ayagoz (town)	93 (22.1)	
	Zharma (town)	92 (21.9)	
	Urzhar (town)	82 (19.5)	
	Kurchatov (town)	62 (14.8)	

**Table 3:** Information sources on food irradiation (N = 420). Familiarity is self-rated; information sources indicate the percentage of respondents who reported hearing about food irradiation from each source.

Source of Information	“Yes” Responses (%)
Heard from TV/Radio	115 (27.4%)
Heard from Internet (web news)	104 (24.8%)
Heard from Social media	152 (36.2%)
Heard from Friends/Family	132 (31.4%)
Heard from Print media	120 (28.6%)
Heard from Authorities/Education	134 (31.9%)

Note: percentages for sources reflect the proportion of the total sample; multiple sources could be selected, so totals exceed 100%.

**Table 4:** Responses to knowledge questions about food irradiation (N = 420). Each row shows a statement and the percentage of respondents answering “True”, “False”, or “Don’t know”. The scientifically correct answer is noted below each statement.

Knowledge Statement	True (%)	False (%)	Don’t Know (%)
“*Irradiated food becomes radioactive*”. (Correct answer: False—irradiated food is not radioactive)	33.3%	19.3%	47.4%
“*Food irradiation kills most bacteria in the food*”. (Correct answer: True—irradiation is effective at pathogen reduction)	33.8%	16.9%	49.3%
“*Once food has been irradiated, it cannot be contaminated again*”. (Correct answer: False—post-irradiation recontamination is possible)	27.4%	19.3%	53.3%
“*Irradiation significantly reduces the nutritional value of food*”. (Correct answer: False—nutrient losses are minimal)	29.3%	20.2%	50.5%
“*The sale of irradiated food is legally permitted in Kazakhstan*”. (Correct answer: True—it is legally allowed under food safety regulations)	30.2%	19.5%	50.2%

**Table 5:** Perceived risk and willingness to purchase irradiated foods, by food category. (“Likely risk” means respondent believes consuming the irradiated food is likely to harm health; “Likely to buy” means respondent would likely purchase/consume the irradiated food if available).

Food Category	“Likely” Harmful (%)	“Neutral/Not Sure” Risk (%)	“Unlikely” Harmful (%)	“Likely” to Buy (%)	“Neutral/Not Sure” Buy (%)	“Unlikely” to Buy (%)
Red Meat	56.2%	20.2%	23.6%	52.6%	30.0%	17.4%
Poultry	56.9%	22.4%	20.7%	48.6%	31.2%	20.2%
Vegetables/Fruit	57.6%	20.0%	22.4%	49.5%	29.5%	21.0%
Infant Food	60.0%	23.1%	16.9%	54.3%	28.8%	16.9%

Percentages may not total 100 in each category due to rounding.

**Table 6:** Pearson correlation matrix among selected variables (N = 420). (Correlation coefficients, r values; none exceed ±0.10 indicating very weak linear relationships except for noted *, the correlation coefficients are less than 0.05).

Variable	Age	Familiarity	Knowledge Score	Risk Perception	Willingness	Trust in Authorities	Naturalness Pref.	Tech Acceptance
Age (years)	1.00							
Familiarity (1–5)	−0.05	1.00						
Knowledge Score (0–5)	0.00	−0.05	1.00					
Risk Perception (avg)	−0.02	−0.01	−0.09	1.00				
Willingness (avg)	−0.07	+0.07	−0.06	+0.08	1.00			
Trust in Authorities	+0.02	−0.10 *	+0.00	−0.01	+0.01	1.00		
Naturalness Preference	−0.02	−0.05	−0.01	−0.07	+0.08	+0.02	1.00	
Tech Acceptance	−0.07	+0.02	+0.01	+0.02	−0.05 *	+0.05	−0.10	1.00

## Data Availability

The original contributions presented in this study are included in the article. Further inquiries can be directed to the corresponding authors.

## Abbreviations

The following abbreviations are used in this manuscript:

SNTS	Semipalatinsk Nuclear Test Site
CIS	Commonwealth of Independent States
kGy	Kilogray
ANOVA	Analysis of Variance
PMT	Protection Motivation Theory
HSD	Honestly Significant Theory
KZT	Kazakhstani Tenge

## References

[1] Rusin T., Villavicencio A.L.C.H., Araújo W.M.C., Faiad C. (2024). How Argentinian Consumers Perceive the Safety of Irradiated Foods. Foods.

[2] Maherani B., Hossain F., Criado P., Ben-Fadhel Y., Salmieri S., Lacroix M. (2016). World Market Development and Consumer Acceptance of Irradiation Technology. Foods.

[3] Chen Q., Cao M., Chen H., Gao P., Fu Y., Liu M., Wang Y., Huang M. (2016). Effects of Gamma Irradiation on Microbial Safety and Quality of Stir Fry Chicken Dices with Hot Chili during Storage. Radiat. Phys. Chem..

[4] Fan X., Niemira B. (2020). Gamma Ray, Electron Beam, and X-Ray Irradiation. Food Safety Engineering.

[5] Lacroix M.L., Jobin M., Latreille B., Nouchpramool K., Gagnon M. (1995). The Effect of Gamma Irradiation on Physical and Nutritional Quality of Penaeus Monodon Shrimps. Radiat. Phys. Chem..

[6] Crawford L.M., Ruff E.H. (1996). A Review of the Safety of Cold Pasteurization through Irradiation. Food Control.

[7] Mostafavi H.A., Fathollahi H., Motamedi F., Mirmajlessi S.M. (2010). Food Irradiation: Applications, Public Acceptance and Global Trade. Afr. J. Biotechnol..

[8] Craft N. (1994). Food Irradiation Is Safe, Says WHO. BMJ.

[9] Resurreccion A.V.A., Galvez F.C.F., Fletcher S.M., Misra S.K. (1995). Consumer Attitudes Toward Irradiated Food: Results of a New Study. J. Food Prot..

[10] Arvanitoyannis I.S. (2010). Consumer Behavior toward Irradiated Food. Irradiation of Food Commodities.

[11] Ablan M., Crawford T.N., Canning M., Robyn M., Marshall K.E. (2025). Consumer Risk Perception of Food Contamination as an Influencer to Purchase Irradiated Ground Beef, Chicken, and Leafy Greens—United States, October 2022. J. Food Prot..

[12] Behrens J., Barcellos M.N., Frewer L., Nunes T.P., Landgraf M. (2009). Brazilian Consumer Views on Food Irradiation. Innov. Food Sci. Emerg. Technol..

[13] Caputo V. (2020). Does Information on Food Safety Affect Consumers’ Acceptance of New Food Technologies? The Case of Irradiated Beef in South Korea under a New Labelling System and across Different Information Regimes. Aust. J. Agric. Resour. Econ..

[14] Eustice R.F., Bruhn C.M. (2007). Consumer Acceptance and Marketing of Irradiated Foods. Food Irradiation Research and Technology.

[15] Maataoui J., Abduljaber M., Khaddor M. (2025). Global Perceptions and Acceptance of Irradiated Food: A Comparative Systematic Review. Ital. J. Food Saf..

[16] Byun M.-W., Oh S.-H., Kim J.-H., Yoon Y., Park S.-C., Kim H.-S., Kim S.-B., Han S.-B., Lee J.-W. (2009). Information Channel Effects on Women Intention to Purchase Irradiated Food in Korea. Radiat. Phys. Chem..

[17] Nayga Jr R.M., Aiew W., Nichols J.P. (2005). Information Effects on Consumers’ Willingness to Purchase Irradiated Food Products. Appl. Econ. Perspect. Policy.

[18] Bruhn C.M. (1995). Strategies for Communicating the Facts on Food Irradiation to Consumers. J. Food Prot..

[19] Hashim I.B., McWatters K., Rimal A., Fletcher S. (2001). Consumer Purchase Behaviour of Irradiated Beef Products: A Simulated Supermarket Setting. Int. J. Consum. Stud..

[20] Stawkowski M.E. (2016). “I Am a Radioactive Mutant”: Emergent Biological Subjectivities at Kazakhstan’s Semipalatinsk Nuclear Test Site. Am. Ethnol..

[21] Nuclear Threat Initiative Semipalatinsk Test Site. https://www.nti.org/education-center/facilities/semipalatinsk-test-site/.

[22] Iwata K., Takamura N., Nakashima M., Alipov G., Mine M., Matsumoto N., Yoshiura K., Prouglo Y., Sekine I., Katayama I. (2004). Loss of Heterozygosity on Chromosome 9q22.3 in Microdissected Basal Cell Carcinomas around the Semipalatinsk Nuclear Testing Site, Kazakhstan. Hum. Pathol..

[23] Larionova N.V., Krivitskiy P.Y., Aidarkhanova A.K., Polevik V.V., Timonova L.V., Monayenko V.N., Turchenko D.V., Lukashenko S.N., Toporova A.V., Aidarkhanov A.O. (2024). Tritium Content in Vegetation Cover at Nuclear Test Locations at the “Sary-Uzen” Site in the Semipalatinsk Test Site. Ecotoxicol. Environ. Saf..

[24] Akhmetova R., Atantayeva B., Abenova G., Karibaev M., Amrina M., Kurbanova N., Pashtetsky V.S., Ivanov A.L., Kudaibergenova I., Sembiyeva L. (2024). The Impact of Nuclear Testing on the Environment: The Case of the Semipalatinsk Nuclear Test Site. Current State, Problems and Prospects for the Development of Agricultural Science, Proceedings of the BIO Web of Conferences, Simferopol, Crimea, 23–27 September 2024.

[25] Aktayev M., Subbotin S., Aidarkhanov A., Aidarkhanova A., Timonova L., Larionova N. (2024). Characterization of Geological and Lithological Features in the Area Proximal to Tritium-Contaminated Groundwater at the Semipalatinsk Test Site. PLoS ONE.

[26] Carlsen T.M., Peterson L.E., Ulsh B.A., Werner C.A., Purvis K.L., Sharber A.C. (2001). Radionuclide Contamination at Kazakhstan’s Semipalatinsk Test Site: Implications on Human and Ecological Health. Hum. Ecol. Risk Assess..

[27] Bauer S., Gusev B.I., Pivina L.M., Apsalikov K.N., Grosche B. (2005). Radiation Exposure Due to Local Fallout from Soviet Atmospheric Nuclear Weapons Testing in Kazakhstan: Solid Cancer Mortality in the Semipalatinsk Historical Cohort, 1960–1999. Radiat. Res..

[28] Kovrigin A.O., Lubennikov V.A., Kolyado I.B., Vikhlyanov I.V., Lazarev A.F., Shoikhet Y.N. (2021). Estimation of cancer incidence in the male population of the Altai Krai affected by the Semipalatinsk nuclear test. Siberian J. Oncol..

[29] Espenbetova M.Z., Bidakhmetova A.M., Krykpayeva A.S., Yespenbetova B.A., Toleutayeva D.M., Serikbayev A.S., Tukinova A.R., Uasheva L.B. (2025). Epidemiology of Thyroid Cancer in Kazakhstan and in Areas Adjacent to the Former Semipalatinsk Nuclear Test Site in 2013-2023. Asian Pac. J. Cancer Preven..

[30] Gusev B.I., Rosenson R.I., Abylkassimova Z.N. (1998). The Semipalatinsk Nuclear Test Site: A First Analysis of Solid Cancer Incidence (Selected Sites) Due to Test-Related Radiation. Radiat. Environ. Biophys..

[31] Svyatova G.S., Zh Abil’Dinova G., Berezina G.M. (2001). The frequency, dynamics, and structure of congenital malformations in populations under long-term exposure to ionizing radiation. Genetika.

[32] Bolegenova N.K., Bekmanov B.O., Djansugurova L.B., Bersimbaev R.I., Salama S.A., Au W.W. (2009). Genetic Polymorphisms and Expression of Minisatellite Mutations in a 3–Generation Population around the Semipalatinsk Nuclear Explosion Test-Site, Kazakhstan. Int. J. Hyg. Environ. Health.

[33] Caffee N. (2020). Between First, Second, and Third Worlds: Olzhas Suleimenov and Soviet Postcolonialism, 1961–1973. Russ. Lit..

[34] International Trade Administration Kazakhstan—Agricultural Sector. https://www.trade.gov/country-commercial-guides/kazakhstan-agricultural-sector.

[35] Follett P.A., Weinert E.D. (2012). Phytosanitary Irradiation of Fresh Tropical Commodities in Hawaii: Generic Treatments, Commercial Adoption, and Current Issues. Radiat. Phys. Chem..

[36] Tong J., Rakovski C., Prakash A. (2015). Phytosanitary Irradiation Preserves the Quality of Fresh Blueberries and Grapes during Storage. HortScience.

[37] Ross R.T., Engeljohn D. (2000). Food Irradiation in the United States: Irradiation as a Phytosanitary Treatment for Fresh Fruits and Vegetables and for the Control of Microorganisms in Meat and Poultry. Radiat. Phys. Chem..

[38] (2015). Food Products. Methods for Determining the Content of Radionuclides.

[39] Kume T., Furuta M., Todoriki S., Uenoyama N., Kobayashi Y. (2009). Status of Food Irradiation in the World. Radiat. Phys. Chem..

[40] Syromyatnikov D., Salimova S., Kolpak E., Mukhametov A. (2024). Analyzing the Impact of Competitiveness Factors on Increasing Grain Production in Kazakhstan and Russia. Heliyon.

[41] Rafihevna Y.S., Asrtovna K.E. (2014). Analysis of Meat Market and Meat Products Market in Kazakhstan. Procedia Soc. Behav. Sci..

[42] Uskelenova A.T., Nikiforova N. (2024). Regional Development of Kazakhstan: Theoretical Premises and Reality. Reg. Sci. Policy Pract..

[43] Cottee J., Kunstadt P., Fraser F.M. (1995). Consumer Acceptance of Irradiated Chicken and Produce in the U.S.A. Radiat. Phys. Chem..

[44] Bruhn C.M. (2007). Enhancing Consumer Acceptance of New Processing Technologies. Innov. Food Sci. Emerg. Technol..

[45] Costa-Font M., Gil J.M., Traill W.B. (2008). Consumer Acceptance, Valuation of and Attitudes towards Genetically Modified Food: Review and Implications for Food Policy. Food Policy.

[46] Gunes G., Deniz Tekin M. (2006). Consumer Awareness and Acceptance of Irradiated Foods: Results of a Survey Conducted on Turkish Consumers. LWT Food Sci. Technol..

[47] Castell-Perez M.E., Moreira R.G., Knoerzer K., Muthukumarappan K. (2021). 2.10—Irradiation and Consumers Acceptance. Innovative Food Processing Technologies.

[48] Loaharanu P. Consumer Acceptance of Irradiated Food. Proceedings of the National Seminar on Acceptance and Trade of Irradiated Foods.

[49] Lima Filho T., Della Lucia S.M., Moulin Lima R., Zacchi Scolforo C. (2015). A Qualitative Study on the Perceptions and Attitudes of Brazilians toward Irradiated Foods. J. Sens. Stud..

[50] Bird W.B. (1998). The Basics of Food Irradiation.

[51] Kilonzo-Nthenge A.K. (2012). Gamma Irradiation for Fresh Produce.

[52] Laminack J., Dainello F., Vestal T.A., Wingenbach G. (2006). Experiential Education Employed to Demystify Food Irradiation as a Viable Technology for Food Industry Professionals. HortTechnology.

[53] Loaharanu P. (1996). Irradiation as a Cold Pasteurization Process of Food. Vet. Parasitol..

[54] Barber N.A., Taylor D.C. (2013). Experimental Approach to Assessing Actual Wine Purchase Behavior. Int. J. Wine Bus. Res..

[55] Tatum D. (2016). The Effect of Labeling on Mitigating Cognitive Biases About Food Irradiation: An Empirical Evaluation of Effects on Consumers’ Attitudes and Purchase Intent. Ph.D. Thesis.

[56] Parrella J., Leggette H., Lu P., Wingenbach G., Baker M., Murano E. (2023). Evaluating Factors Explaining U.S. Consumers’ Behavioral Intentions toward Irradiated Ground Beef. Foods.

[57] Van Dijk H. (2010). Consumer Responses to Risk-Benefit Information About Food.

[58] Sapp S.G. (2003). A Comparison of Alternative Theoretical Explanations of Consumer Food Safety Assessments. Int. J. Consum. Stud..

[59] Sapp S.G., Downing-Matibag T. (2009). Consumer Acceptance of Food Irradiation: A Test of the Recreancy Theorem. Int. J. Consum. Stud..

[60] Ueland Ø., Gunnlaugsdottir H., Holm F., Kalogeras N., Leino O., Luteijn J.M., Magnússon S.H., Odekerken G., Pohjola M.V., Tijhuis M.J. (2012). State of the Art in Benefit–Risk Analysis: Consumer Perception. Food Chem. Toxicol..

[61] Youn H.-J., Lee J.-H. (2020). Investigation of the Possibility of Applying Protection Motivation Theory in Consumers’ Changes by Fipronil Egg Contamination. J. Korean Diet. Assoc..

[62] Zhu Y., Wen X., Chu M., Sun S. (2022). Consumers’ Intention to Participate in Food Safety Risk Communication: A Model Integrating Protection Motivation Theory and the Theory of Reasoned Action. Food Control.

[63] Henson S., Cranfield J., Herath D. (2010). Understanding Consumer Receptivity towards Foods and Non-prescription Pills Containing Phytosterols as a Means to Offset the Risk of Cardiovascular Disease: An Application of Protection Motivation Theory. Int. J. Consum. Stud..

[64] Choi Y., Kim J., Han E. (2016). Analysis of Food Irradiation Education for Elementary, Middle, and High School Students for Three Years in South Korea. Nutr. Res. Pract..

[65] Roberts P.B. (2016). Food Irradiation: Standards, Regulations and World-Wide Trade. Radiat. Phys. Chem..

[66] Fox J.A. (2002). Influences on Purchase of Irradiated Foods. Food Technol..

[67] Zienkewicz L.S.H., Penner K.P. (2004). Consumers’ Perceptions of Irradiated Ground Beef after Education and Product Exposure. Food Prot. Trends.

[68] Cox D.N., Evans G. (2008). Construction and Validation of a Psychometric Scale to Measure Consumers’ Fears of Novel Food Technologies: The Food Technology Neophobia Scale. Food Qual. Prefer..

[69] Frewer L.J., Bergmann K., Brennan M., Lion R., Meertens R., Rowe G., Siegrist M., Vereijken C. (2011). Consumer Response to Novel Agri-Food Technologies: Implications for Predicting Consumer Acceptance of Emerging Food Technologies. Trends Food Sci. Technol..

[70] Cardello A.V. (2003). Consumer Concerns and Expectations about Novel Food Processing Technologies: Effects on Product Liking. Appetite.

[71] Wheeler T., Shackelford S., Koohmaraie M. (1999). Trained Sensory Panel and Consumer Evaluation of the Effects of Gamma Irradiation on Palatability of Vacuum-Packaged Frozen Ground Beef Patties. J. Anim. Sci..

[72] Luchsinger S., Kropf D., Iv E.C., Zepeda C.G., Hunt M., Stroda S., Hollingsworth M., Marsden J., Kastner C. (1997). SENSORY ANALYSIS OF IRRADIATED GROUND BEEF PATTIES AND WHOLE MUSCLE BEEF 1. J. Sens. Stud..

[73] Risvik E. (1986). Sensory Evaluation of Irradiated Beef and Bacon. J. Sens. Stud..

[74] Zhang M., He L., Li C., Yang F., Zhao S., Liang Y., Jin G. (2020). Effects of Gamma Ray Irradiation-Induced Protein Hydrolysis and Oxidation on Tenderness Change of Fresh Pork during Storage. Meat Sci..

[75] Bearth A., Siegrist M. (2019). “As Long as It Is Not Irradiated”–Influencing Factors of US Consumers’ Acceptance of Food Irradiation. Food Qual. Prefer..

